# Acute trophoblastic pulmonary embolism during conservative treatment of placenta accreta: case report and review of literature

**DOI:** 10.1186/s40001-015-0185-6

**Published:** 2015-11-14

**Authors:** Qiu-ming Wang, Hui-li Liu, Qun Dang

**Affiliations:** Department of Gynecology, The Second Affiliated Hospital of Zhengzhou University, Zhengzhou, 450000 China; Department of Gynecology, Henan Provincial People’s Hospital, Zhengzhou, 450000 China

**Keywords:** Trophoblastic pulmonary embolism, Allergic shock, Placenta accreta, Conservative treatment

## Abstract

**Background:**

Placenta accreta is a rare obstetric condition but can lead to life-threatening complications that was mainly diagnosed in the third trimester. We present a case of acute trophoblastic pulmonary embolism (PE) during conservative treatment of placenta accreta.

**Case presentation:**

A 24-year-old patient who delivered vaginally at 40^+4^ weeks gestation. The placenta was retained despite the use of oxytocics and attempts of manual removal. Conservative management including uterine arteria embolism, hysteroscopic resection and mifepristone was used but failed and finally the patient died from acute trophoblastic PE and allergic shock when infusing povidone-iodine into her uterine cavity.

**Conclusion:**

Although conservative treatment of placenta accreta can retain reproductive potential and trophoblastic PE is rare, clinicians should consider hysterectomy when conservative treatment failed and infusion of povidone-iodine or other liquid into the cavity should be prohibited.

## Background

Placenta accreta indicates deep attachment of the placenta to myometrium due to the absence of decidua basalis [1]. The optimal management of this condition remains controversial. Although hysterectomy is a choice for retained placenta, leaving the placenta in situ with conservative treatment such as uterine arteria embolism, mifepristone, methotrexate and hysteroscopic resection can also be undertaken when there is no hemorrhage and the patient want to preserve her fertility. Conservative management also has some risk such as hemorrhage, infection, failure and subsequent hysterectomy, and even death [2].

We present a unique case of death caused by acute trophoblastic PE and anaphylactic shock during conservative treatment of placenta accreta. To our knowledge, this is the first report about trophoblastic PE during conservative management of placenta accreta.

## Case presentation

**Fig. 1:**
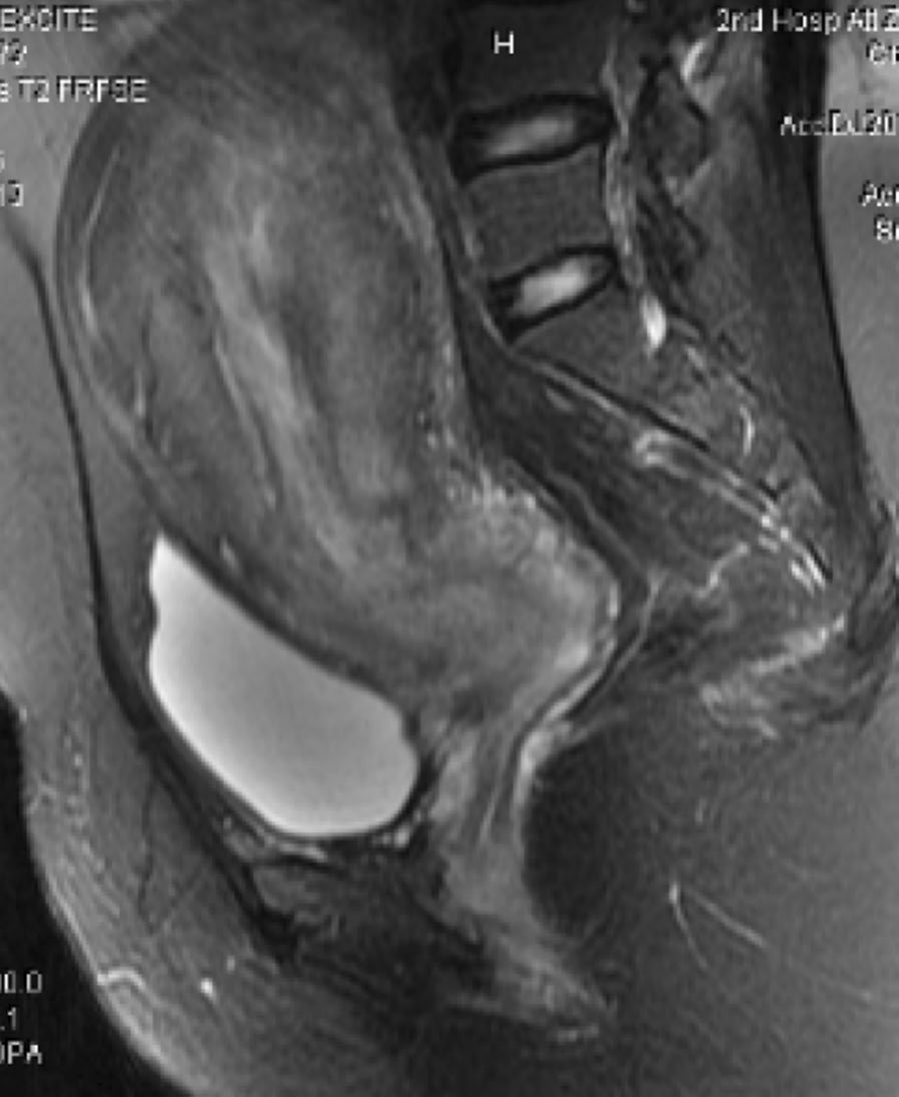
MRI demonstrates segment of placenta extending outside of the visible myometrium

**Fig. 2:**
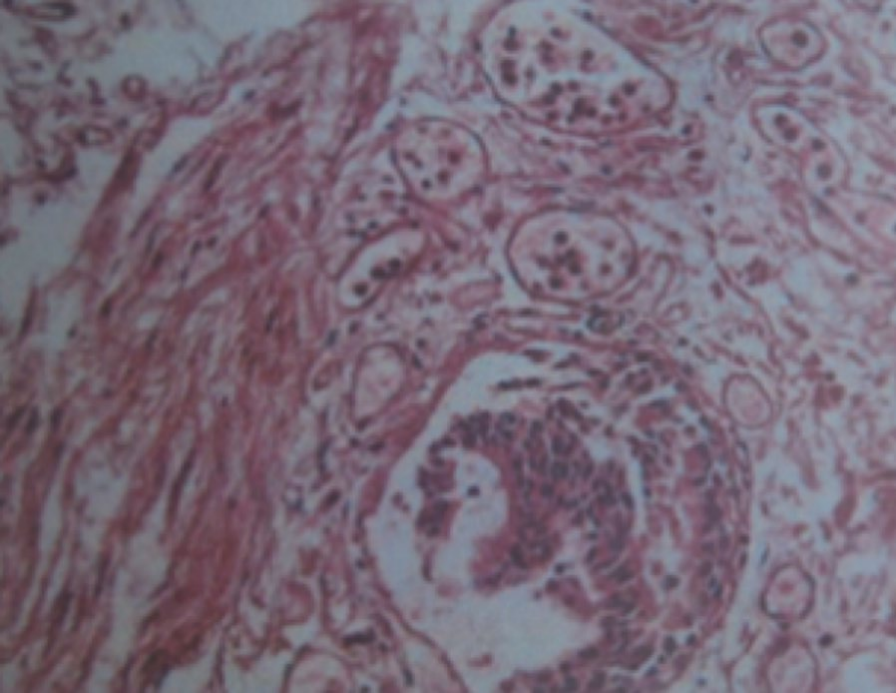
Histopathological picture showing that trophoblastic emboli can be found in the pulmonary interstitial vessels

**Fig. 3:**
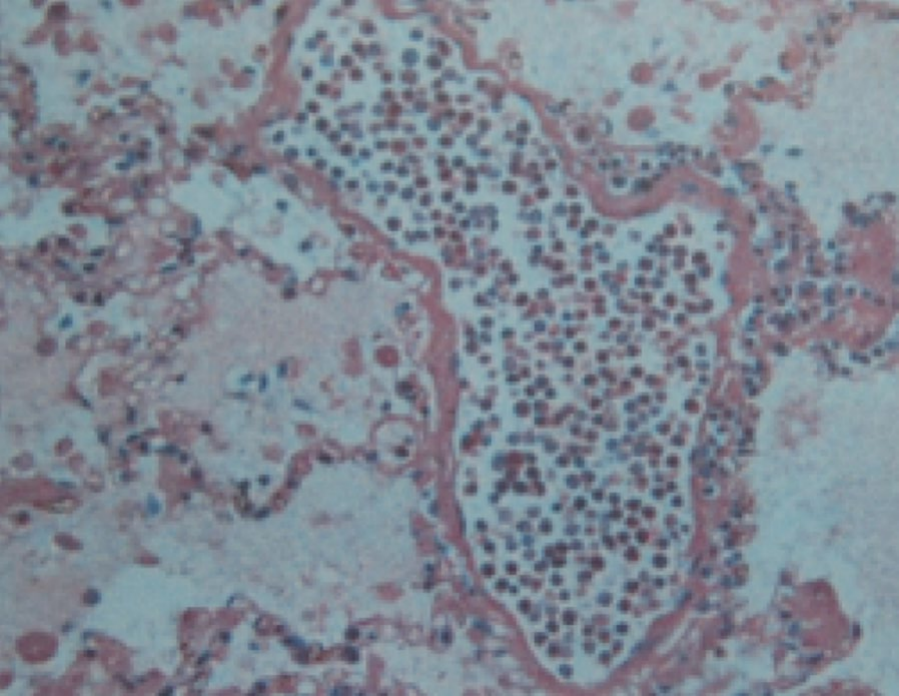
Histopathological picture showing that a lot of eosinophils can be found in pulmonary interstitial vessels

A woman with a history of one spontaneous miscarriage of 7-week gestation without curettage or other treatment had a vaginal delivery at 40^+4^ weeks gestation. The placenta was retained in the cavity despite the use of oxytocics. Manual removal of the placenta was attempted, but unsuccessful. Emergency ultrasound scan revealed placenta tissue measuring 165 × 130 × 94 mm^3^ without a clear boundary with myometrium. Magnetic resonance imaging (MRI) confirmed placenta accreta the next day (Fig. 1). Her pre-delivery hemoglobin concentration was 11.9 g/dL and the estimated blood loss was 200 mL without active hemorrhage. She wanted to keep her uterus and declined hysterectomy, so uterine arterial embolism was performed in case of massive hemorrhage. After embolization, her general condition was fine except mild pelvic pain. Prophylactic intravenous injections of Cefazolin Sodium Pentahydrate 1.0 g and Ornidazole 0.5 g, 12 hourly were used to decrease the chance of infection. 3 days later her body temperature raised to 38.3 °C, laboratory result on C reactive protein was >200 mg/L and white blood cell count was 18.5 × 10^9^/L. Escherichia coli was isolated from vagina secretion. Conservative treatment needed to be abandoned but she still declined hysterectomy. Cefazolin Sodium Pentahydrate was changed to Cefoperazone-Sulbactam 3 g, 12 hourly according to the drug-sensitive test. Her general condition became better and she was transferred to gynecology. To accelerate the placenta elimination, hysteroscopic resection was undertaken 9 days after her delivery. Placenta could be seen everywhere in the cavity from the hysteroscope. The surgery had to terminated 90 min later to avoid water intoxication. About 6 × 6×1 cm^3^ of placenta was resected. Mifepristone was used 25 mg orally daily to promote necrosis of trophoblastic as her serum β-hCG was still 2608 mIU/ml. The next day after hysteroscopy her body temperature raised to 40.3 °C. Blood culture was negative but Escherichia coli could still be found in vagina secretion. Antibiotics were changed to Amikacin 0.6 g q12 h and Cefoxitin 2.0 g q8 h according to drug-sensitive test. Purulent secretion and necrotic tissue can be seen in the cervix and cavity during gynecological examination. Povidone-iodine was infused into her cavity to wash out the purulent secretion and necrotic tissue with a double cavity flexible pip with her permission. During this procedure, the second time the patient immediately experienced dyspnea and lost consciousness soon after about 15 mL povidone-iodine was infused into her cavity. The clinical picture suggested acute respiratory distress syndrome. Cardiopulmonary resuscitation was performed immediately. However, despite all efforts, the patient died. Written consent was obtained from her relatives and autopsy was performed to investigate the underlying pathological condition. Autopsy revealed that trophoblastic PE (Fig. 2) and anaphylactic shock (Fig. 3) were the causes of her death.

## Discussion

Commonly risk factors for placenta accreta are previous cesarean section, dilatation and curettage, placenta previa, previous uterine injury or surgery [3]. As the cesarean section rate and artificial abortion rates are rising, the placenta accreta rate is also increasing [4], especially in China. In some part of mainland China, the cesarean section rate is as much as 54.9 %, the highest in the world [5]. In contrast to many reports, our patient had no risk factor mentioned above.

Ultrasound and MRI have been described to diagnose placenta accreta in literature. However, prenatal diagnosis of placenta accreta remains a challenge. Most of placenta accreta was diagnosed in the third stage of labor [6]. One prospective population-based study of women diagnosed with placenta accreta found that only less than 50 % (66/133) were suspected antenatally. Antenatally diagnosis of placenta accreta reduced the ratio of hemorrhage and blood transfusion with statistical significance. So antenatally diagnosis is very important for placenta accreta [7]. In our case, prenatal ultrasound also did not diagnose the placenta accreta.

Placenta accreta morbidity is mainly caused by hemorrhage, especially when surgical attempts are used to remove the placenta, which can cause intraoperative and postoperative maternal morbidity, large-volume blood transfusions, ureteral damage, intraabdominal infection [2]. So surgical attempts to remove placenta should be avoided when placenta accreta is suspected. The options for managing placenta accreta include hysterectomy and conservative treatment. If the placenta accreta was diagnosed before delivery or during cesarean section, cesarean hysterectomy is generally considered the standard treatment for placenta accreta. Subsequent hysterectomy after vaginal delivery is also appropriate when massive hemorrhage is threatening life. Conservative treatment may be applied for some women who want to keep their uterus when there is no hemorrhage.

There is no standard conservative treatment option for placenta accreta. Conservative treatment includes uterine artery embolization, methotrexate, mifepristone and hysteroscopic resection. Uterine artery catheterization can reduce risk of massive hemorrhage in the management of placenta accreta [8]. Methotrexate has been used to promote the necrosis and resorption of placenta. However, methotrexate has some serious side effects, women unable to breastfeed and a maternal death related to the use of methotrexate has been reported [2]. Royal College of Obstetricians and Gynaecologists noted that methotrexate has little benefit in enhancing placental resorption [9]. Mifepristone has been successfully used in placenta accreta without any complication [10]. Leaving the placenta in situ after delivery can delay placental expulsion or resorption for weeks or even months. Resection of persistent retained placenta tissue by hysteroscopy can shorten this period and reduce the risk for infection and bleeding [11]. One large series on the conservative treatment of placenta accreta includes uterotonic drugs (oxytocin or sulprostone or both), prophylactic antibiotic therapy, methotrexate, pelvic arterial embolization. The success rate of this study was 78.5 % (131/167) and the remaining 36 women had hysterectomy [2]. In our case, although three conservative treatments were applied to her, the serum β-hCG was still 2890 mIU/mL before death. Her body temperature and laboratory result suggested she got an infection. Those indicate that conservative treatments were not appropriate for her and hysterectomy should be applied immediately but she declined.

PE is defined as embolisation to the pulmonary circulation by thrombus, adipocytes, amniotic fluid, tumor, bacteria, or gas, but trophoblastic tissue is very rare. Unlike other kind of embolus, the presence of trophoblastic tissue in the maternal circulation is not considered abnormal [12]. Between 30 and 80 % of obstetric patients have trophoblastic cells in their peripheral circulation. However, the quantity of trophoblastic tissue found in pulmonary vessels indicates that trophoblastic PE led to maternal death. The diagnosis of trophoblastic PE is based on both clinical information and pathological outcomes [13]. Ikarashi et al. [14] identified two histopathological patterns of trophoblastic PE: one composed of intact trophoblastic cells similar to those seen in chorionic villi and identified easily by H&E staining; the other consisted of amorphous and fragmented trophoblastic cells that were more difficult to identify by routine staining.

Trophoblastic PE was first described by Schmorlin 1893 in a postmortem study of eclamptic patients [15]. Most of trophoblastic PE happens in gestational trophoblastic disease, during cesarean section and curettage. Garner et al. [16] report on trophoblastic PE in a young woman following abdominal hysterectomy for invasive gestational trophoblastic disease. Fortunately, she got complete recovery after 72 h with the treatment of supportive measures. Tews et al. [13] report on sudden death in a young woman following cesarean section and histological investigation revealed trophoblastic cells within the lumen of small-sized pulmonary vessels and thought that microscopic injuries during decompression punctures might have favored the deportation of trophoblastic tissue into the maternal circulation. Seiryu et al. [17] report on trophoblastic PE in a young woman during the curettage procedures of 7-week gestation and thought that the trophoblastic PE was caused by direct access of trophoblastic tissues to the uterine veins draining the maternal intervillous space during the curettage procedures.

To our knowledge, this is the first case of trophoblastic PE to occur during conservative treatment of placenta accreta. The pathogenesis of emboli is far more complex than mechanical obstruction of vascular, especially nonthrombotic emboli. Nonthrombotic emboli such as trophoblastic cells may also lead to a severe inflammatory reaction [18]. In our case, anaphylactic shock also accompanied the trophoblastic PE.

Povidone-iodine is commonly used in infectious wound. We infused povidone-iodine into her cavity with the purpose of washing out necrotic tissue and purulent secretion to reduce infection. There was no active hemorrhage so we did not predict that povidone-iodine or some other things could get into her circulation. May be the reason for trophoblastic PE was that as three conservative treatments had been applied to her, the retained placenta is necrotic and loosely adhered to the myometrium. The sudden introduction of povidone-iodine may have detached part of the placenta and exposing a blood vessel; the trophoblastic cells entered the blood circulatory system and the trophoblastic PE happened. Therefore, infusion of povidone-iodine or some other liquid to the cavity during conservative treatment of placenta accreta should be avoided.

## Conclusion

We present a unique case of acute trophoblastic PE during conservative treatment of placenta accreta in a 24-year-old female. Careful evaluation of this rare complication and painful lesson may provide us some experience in the treatment of placenta accreta. This case reminds us that hysterectomy should be employed when conservative treatment of placenta accreta fails to avoid severe complications. Infusion of povidone-iodine or other liquid to the uterine cavity during conservative treatment of placenta accreta should be avoided.

## Consent

Written informed consent was obtained from the patient for publication of this Case report and any accompanying images. A copy of the written consent is available for review by the Editor-in-Chief of this journal.

## Abbreviations

PE: pulmonary embolism
MRI: magnetic resonance imaging
